# Gene Expression Analysis Reveals Novel Shared Gene Signatures and Candidate Molecular Mechanisms between Pemphigus and Systemic Lupus Erythematosus in CD4^+^ T Cells

**DOI:** 10.3389/fimmu.2017.01992

**Published:** 2018-01-17

**Authors:** Tanya Sezin, Artem Vorobyev, Christian D. Sadik, Detlef Zillikens, Yask Gupta, Ralf J. Ludwig

**Affiliations:** ^1^Department of Dermatology, University of Lübeck, Lübeck, Germany; ^2^Lübeck Institute of Experimental Dermatology (LIED), University of Lübeck, Lübeck, Germany

**Keywords:** autoimmunity, gene expression analysis, weighted gene co-expression analysis, pemphigus, systemic lupus erythematosus, CD4^+^ T cells

## Abstract

Pemphigus and systemic lupus erythematosus (SLE) are severe potentially life-threatening autoimmune diseases. They are classified as B-cell-mediated autoimmune diseases, both depending on autoreactive CD4^+^ T lymphocytes to modulate the autoimmune B-cell response. Despite the reported association of pemphigus and SLE, the molecular mechanisms underlying their comorbidity remain unknown. Weighted gene co-expression network analysis (WGCNA) of publicly available microarray datasets of CD4^+^ T cells was performed, to identify shared gene expression signatures and putative overlapping biological molecular mechanisms between pemphigus and SLE. Using WGCNA, we identified 3,280 genes co-expressed genes and 14 co-expressed gene clusters, from which one was significantly upregulated for both diseases. The pathways associated with this module include type-1 interferon gamma and defense response to viruses. Network-based meta-analysis identified RSAD2 to be the most highly ranked hub gene. By associating the modular genes with genome-wide association studies (GWASs) for pemphigus and SLE, we characterized IRF8 and STAT1 as key regulatory genes. Collectively, in this *in silico* study, we identify novel candidate genetic markers and pathways in CD4^+^ T cells that are shared between pemphigus and SLE, which in turn may facilitate the identification of novel therapeutic targets in these diseases.

## Introduction

Pemphigus is a rare autoimmune bullous dermatosis, clinically characterized by intraepidermal blistering of the skin and/or mucous membranes. Immunologically, pemphigus is characterized by autoantibodies directed against desmosomal and non-desmosomal adhesion molecules expressed in the skin and mucosa. Binding of the pathogenic autoantibodies in the skin leads to dissociation of adjacent keratinocytes and formation of blisters. Based on the clinical presentation and the specificity of the anti-desmoglein (Dsg) autoantibodies, pemphigus is classified into two main forms, pemphigus vulgaris (PV), with autoantibodies targeting Dsg3, and in some cases also Dsg1, and pemphigus foliaceus (PF), with autoantibodies targeting Dsg1 (1). The association of pemphigus with connective tissue diseases such as systemic lupus erythematosus (SLE) has been previously noted on a case report/case series basis (2, 3). In line, pemphigus autoantibodies and antinuclear autoantibodies, one immunological hallmark of SLE (4), coexist in healthy blood donors (5). However, the molecular mechanism remains unknown. The co-occurrence of pemphigus and SLE can suggest a common network of multifunctional genes and pathways. Alternatively, it can be altogether serendipitous. Due to the complexity of such a system, weighted gene co-expression network analysis (WGCNA) can serve as a comprehensive tool for identifying gene clusters of correlating and connected shared genes (6, 7). This approach has been previously successfully applied in various biological contexts to identify regulatory genes and networks associated with multiple disease phenotypes (8–11).

Systemic lupus erythematosus and pemphigus are characterized by the production of autoantibodies and traditionally classified as B-cell-mediated autoimmune diseases. Compelling evidence has, however, shown that autoreactive helper-T lymphocytes are crucial in pathogenicity of both diseases by regulating B cells response and promoting autoantibodies production (12–15). Thus, studying gene expression networks within the CD4^+^ T-cell population is not only essential for understanding the underlying pathophysiology but also for identifying predictive biomarkers and establishment of novel therapeutic targets for these diseases.

Using publically available gene expression data from NCBI GEO database, we investigated gene co-expression networks of CD4^+^ T cells obtained from pemphigus (PV as well as PF) and SLE patients (16). Our analysis revealed 14 distinct modules containing 3,280 co-expressed genes between the two diseases. Two out of 14 modules were found significantly upregulated: one in PF and SLE, and the other in PV. We further identified biological pathways such as “type I interferon signaling pathway” and “defense response to virus” using KEGG database, to be enriched in disease-associated modules. To the best of our knowledge, this is the first study applying a systems biology approach to identify shared molecular mechanisms between pemphigus and SLE.

## Materials and Methods

### Data Collection

All the data for the analysis were collected by searching expression databases such as NCBI GEO and Array Express for CD4^+^ T cells for pemphigus and SLE (17, 18). The datasets from other tissues or cell type were discarded. Also, the datasets, which did not have raw data files, were discarded from the downstream analysis. Two datasets, one for pemphigus (GSE53873) and one for SLE (GDS4185), were included in this study. The covariate information available for the patients is summarized in Table S1 in Supplementary Material. Altogether 46 samples (4 PV, 15 PF, 13 SLE, and 14 healthy controls) were used in the analysis.

**Figure 1:**
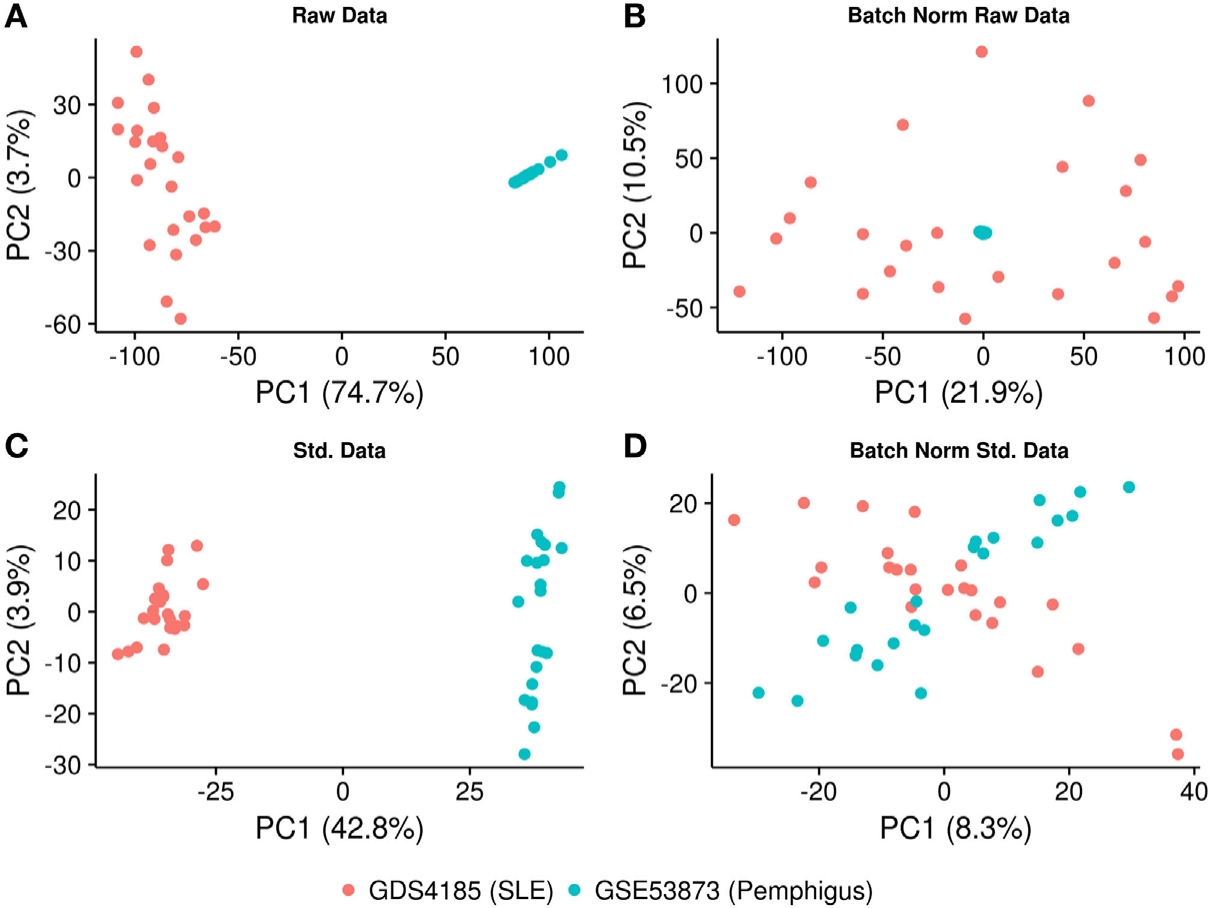
PCA plot illustrating the normalization procedure. **(A)** PCA plot showing clustering of the samples based on the gene expression profiling, before and **(B)** after batch correction on raw data. **(C)** PCA plot showing clustering of the samples after using identical background correction and normalization methods, before and **(D)** after batch correction. The *X*- and *Y*-axes represent the first and the second principal components and the associated percentage of variation.

To avoid a potential bias that could be introduced by obtaining two separate microarray datasets, the deposited gene expression data were directly used for batch normalization. The expression profiles were log2 transformed and batch normalization was done using “sva” and “combat” functions in SVA R package (19). The effect of normalization was investigated by principal component analysis using the R-based “prcomp” function. Since batch normalization still produced biased results (Figure 1), the raw files were preprocessed again and an additional normalization step was performed. In detail, raw gene expression profiles were deduced from text files (Codelink array) using Codelink R package (20). Using the same package, first, the background was corrected with the “normexp” method and then normalized by the “cyclicloess” method. For Affymetrix data, raw gene expression for each sample was derived using R Affy package (21). The background correction was performed by “backgroundCorrect (method = ‘normexp’)” and cyclic normalization was performed on log2 expression values using limma R package (22). All the probes from each of the microarray platforms were filtered out for significant low expression/variation (*P* < 0.05) using the “varianceBasedfilter” function from DCGL R package (23). The remaining probes were mapped to Ensembl gene identifiers and probes’ expression was collapsed to gene-level expression using “collapseRows” function with default parameters in WGCNA R package (24). Consequently, batch normalization and statistical analysis were performed on the overlapping genes between two platforms using “combat” and PCA analyses, respectively (25). The data were further investigated for the presence of confounding effects such as clinical form of the disease (generalized vs. localized) and treatment group (predisnome vs. untreated) for pemphigus dataset (GSE53873) using anosim function with 999 permutations in vegan R package (26).

### Co-Expression Networks

Co-expression modules were generated using WGCNA R package. A signed weighted adjacency matrix of pair-wise connection strengths (bicor correlation) was constructed using the soft-threshold approach with a scale-independent topological power β = 6. For a gene, the connectivity was defined as the sum of all connection strengths with all other genes. Genes were aggregated into modules by hierarchical clustering and refined by the dynamic tree cut algorithm. Thereafter, module eigenvalues were calculated. The eigenvalue is the first principal component of the gene expression profile within a module, representing average module expression profile (27). The statistical significance (*P* < 0.05) of module eigenvalues among the groups was accessed by Kruskal–Wallis test. Modular hub gene candidates were identified by correlating the gene expression with its module eigenvalues (“chooseTopHubInEachModule” function in WGCNA). To generate the causal network within a module, the C3NET R package was used (28). The algorithm uses mutual information theory to construct gene networks from gene expression data. The final network was generated using “c3net” function with default setting. A gene–gene interaction was considered to be significant if α < 0.05.

### Functional Characterization of a Module

To investigate known gene–gene interactions, we used the INMEX web server (29). All genes within a specific module were queried, and a minimum network connecting all genes within this module was obtained. The hub gene candidates from this analysis were defined by their degree of interactions. Gene ontology terms, enriched KEGG pathways, and transcription factor binding sites for each module were obtained using David web server. Thereafter, all the mapped genes and reported genes to the disease-associated loci were selected from genome-wide association study (GWAS) catalog. The selected genes and modular genes were connected to each other based on known gene–gene interactions (INMEX web server). Only the direct interactions between the modular genes and GWAS genes were considered. Gene–gene interactions were visualized using Cytoscape software and figures were generated using R programing language. Intermediate gene conversions and data formatting were done using Perl programing language (30).

## Results

### Data Selection and Normalization

Microarray data were obtained for peripheral CD4^+^ T-cell samples from 19 pemphigus patients (4 PV; 15 PF), 13 SLE patients, and 14 healthy controls from NCBI GEO and EBI Array Express (GSE53873; GDS4185). Altogether, our dataset included 46 samples derived from Codelink and Affymetrix arrays. Only datasets comprising raw files were included in the downstream meta-analysis. Therefore, we excluded samples GSE4588 and GSM260948 from our analysis.

To implement the co-expression network analysis, we standardized and batch-normalized the datasets. We collected common probes across the two chip-arrays. The CodeLink Human Whole Genome Bioarray from GE Healthcare consisted of 54,359 probes, while the Affymetrix Human Genome U133A array consisted of 22,283 probes. We converted these probes to ensemble gene identifiers using ensemble biomart and found that 12,980 genes were common between the two platforms. Consequently, the datasets were merged based on the expression of common genes and “combat” and “sva” (SVA R package) functions were applied to remove the batch effect. Our results show that while the Affymetrix samples were distributed uniformly among the principal components, the data generated from the CodeLink array still clustered together (Figures 1A,B), suggesting that the dataset was not properly normalized and required further optimization. To further optimize the datasets, we used the “normexp” method for background correction and “cyclicloess” on log2 transformed values. Additionally, each dataset was separately filtered for low expressing/varying probes, as well as multiple probes were collapsed for each gene. Briefly, 18,038 probes representing 12,980 genes were identified in the CodeLink dataset. These probes were filtered for low variation and collapsed to generate 5,646 gene expression profiles. Similarly, the Affymetrix gene chip consisted of 20,366 probes representing 12,980 genes. These probes were filtered and collapsed, resulting in 6,073 gene expression profiles. Overall, the overlap between the two datasets consisted of 3,280 gene expression profiles, which were further used in the downstream analysis. After applying the batch effect normalization “combat” algorithm, we observed that the samples were distributed among first principal component with only 8.3% variation explained by the first component (Figures 1C,D). We also analyzed confounding effects by stratifying the dataset for different covariates. We found no significant differences for covariate generalized vs. localized (*P* = 0.402) and prednisone treated vs. untreated (*P* = 0.596) for pemphigus samples. No covariate information was available for SLE samples (Figure S1 in Supplementary Material).

### Detection of Co-Expression Modules Related to Pemphigus and SLE

**Figure 2:**
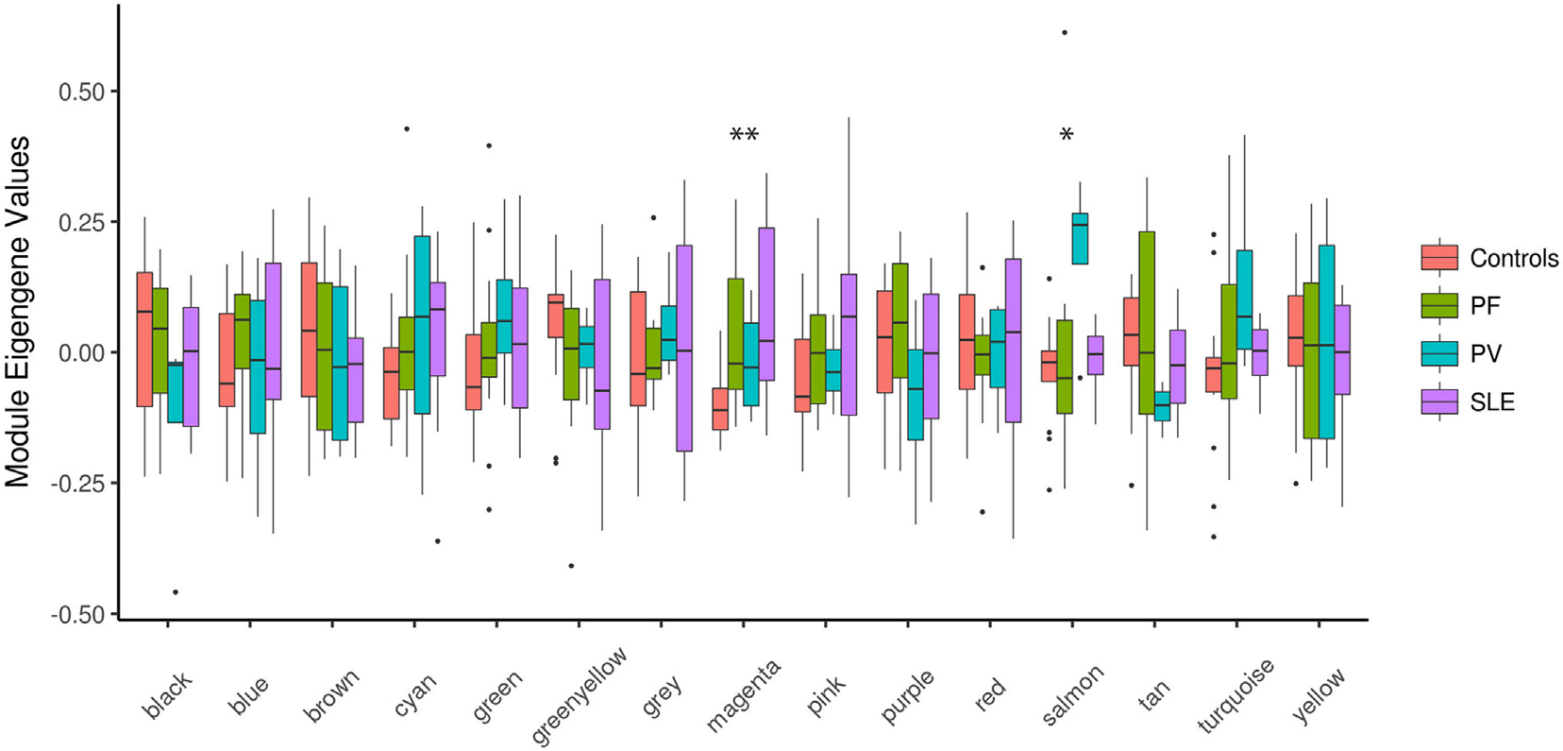
Boxplots of eigengene values across modules. Boxplots depicting different identified modules on the *X*-axis and the corresponding module eigengene values for each group of samples on the *Y*-axis. The significance among the groups was calculated using Kruskal–Wallis test. *P < 0.05; **P < 0.01. PF, pemphigus foliaceus; PV, pemphigus vulgaris; SLE, systemic lupus erythematosus.

Next, we set out to identify system-level similarity between pemphigus and SLE. Therefore, we applied WGCNA, aiming to identify gene modules that are co-expressed between pemphigus and SLE samples, and that are likely to be involved in common pathways. The major advantage of using such an approach is that it alleviates the multiple-testing problem that is inherent to microarray datasets. Using WGCNA, we identified 14 modules of co-expressed genes for 3,280 highly expressed and varying gene expression profiles, which are represented by different color codes (Figure 2; Figure S1, Data Sheet 1 in Supplementary Material). Two out of 14 modules showed differences between control and disease samples. The module “magenta” was significantly upregulated for both PF (*P* = 0.005) and SLE (*P* = 0.016) in comparison to healthy controls, and the module “salmon” was specifically upregulated only in PV (*P* = 0.034) (Figure 2).

### Biological Pathways in the PF- and SLE-Associated Module “Magenta”

**Figure 3:**
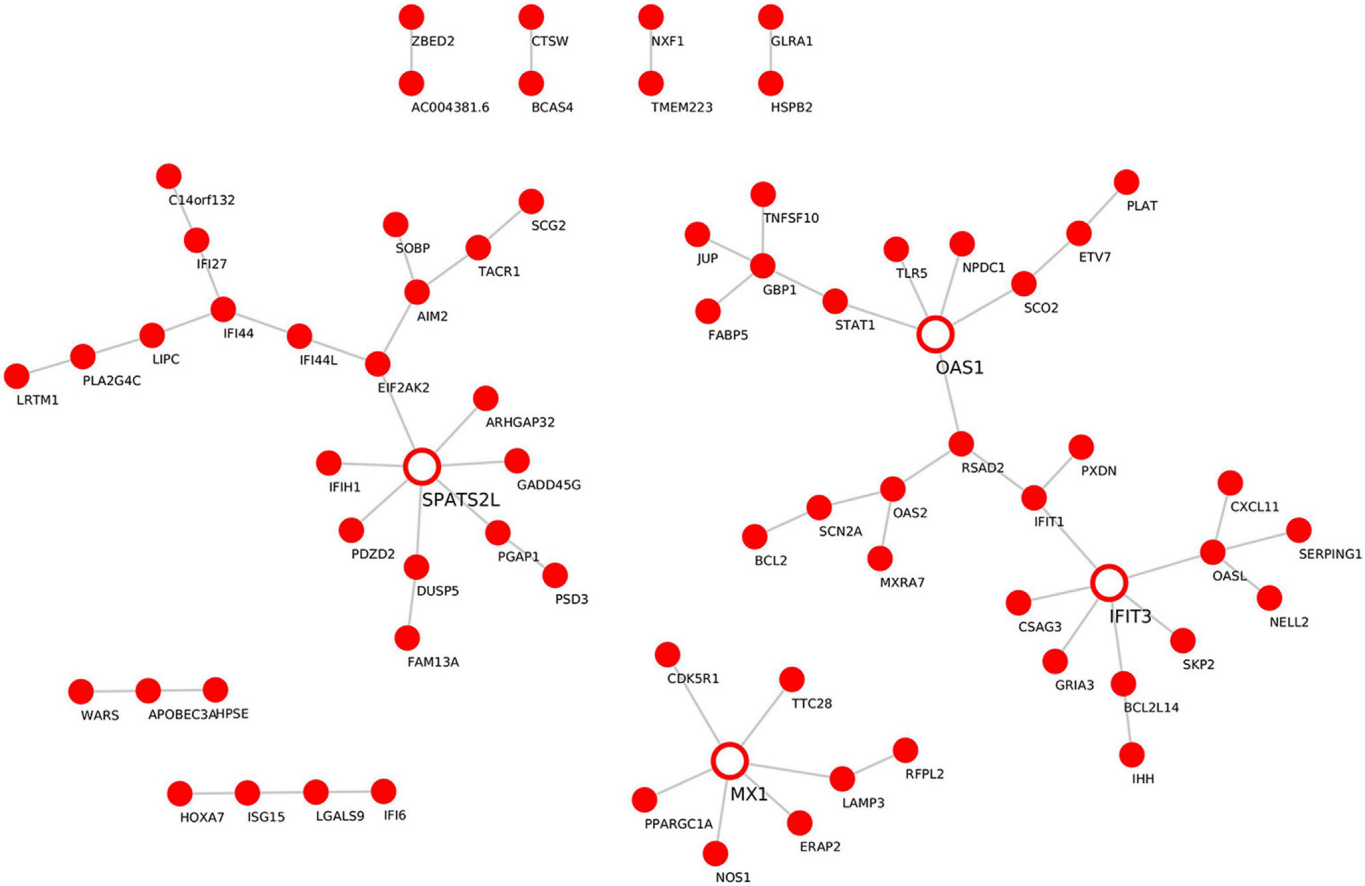
Gene–gene interaction network for the “magenta” module. *De novo* network generated by C3NET algorithm for the “magenta” module. The figure shows statistically significant (α < 0.05) edges predicted by the algorithm. Fully colored nodes represent the “magenta” module-associated genes. Empty nodes represent the regulatory genes (degree ≥ 5).

Module “magenta” consisted of 74 genes and, compared with controls, was significantly upregulated in PF and SLE. To investigate different known mechanisms associated with this module, we performed gene ontology analysis using DAVID database (31). We found that this module was, among others, enriched in biological processes such as “type I interferon signaling pathway” (P.adj = 6.4E−11), “defense response to virus” (P.adj = 2.7E−10), and “cytokine-mediated signaling pathway” (P.adj = 1.3E−7) (Table 1). This module was also enriched in KEGG pathways, including “measles” (P.adj = 2.3E−4), “influenza A” (P.adj = 2.7E−4), and “herpes simplex infection” (P.adj = 1.3E−3). On the basis of statistical module membership and eigengenes value, we identified s-adenosyl methionine domain containing 2 (RSAD2) gene as the most highly ranked hub gene for this module. To identify subnetworks and statistical interactions within the modules we used the “c3net” algorithm. The “c3net” algorithm investigates the direct physical interaction for gene expression data, further providing putative mechanisms within a module and characterizing its key regulating genes (9). We found 2’-5’-oligoadenylate synthetase 1 (OAS1), MX dynamin-like GTPase 1 (MX1), interferon-induced protein with tetratricopeptide repeats 3 (IFIT3), and spermatogenesis-associated serine-rich 2 like (SPATS2L) genes as master regulator genes of the module (degree ≥ 5) (Figure 3). Moreover, to further explore known gene–gene interactions among the genes in “magenta” module, we used the INMEX web server (32). We were specifically interested in examining “minimum interaction networks.” In this type of networks, a minimum number of genes are required to connect all the nodes to a given set of genes. Using this approach, we further derived additional regulators such as junction plakoglobin (JUP), B-cell CLL/lymphoma 2 (BCL2), ISG15 ubiquitin-like modifier (ISG15), STAT1, S-phase kinase-associated protein 2 (SKP2), and eukaryotic translation initiation factor 2 alpha kinase 2 (EIF2AK2) (Figure S2 in Supplementary Material).

**Table 1:** Gene ontology and enriched KEGG pathways for “magenta” and “salmon” modules.

Module	Category	Term	*P*-value	Benjamini
Magenta	UP_KEYWORDS	Antiviral defense	1.18273E−16	1.84297E−14
	UP_KEYWORDS	Immunity	1.22704E−13	1.01824E−11
	GOTERM_BP_DIRECT	GO:0060337~type-I interferon signaling pathway	9.37804E−14	6.3981E−11
	UP_KEYWORDS	Innate immunity	3.82091E−12	2.11426E−10
	GOTERM_BP_DIRECT	GO:0051607~defense response to virus	7.83394E−13	2.6713E−10
	GOTERM_BP_DIRECT	GO:0045071~negative regulation of viral genome replication	1.21675E−10	2.76607E−08
	GOTERM_BP_DIRECT	GO:0009615~response to virus	2.90413E−10	4.95154E−08
	GOTERM_BP_DIRECT	GO:0019221~cytokine-mediated signaling pathway	9.178E−10	1.25188E−07
	KEGG_PATHWAY	hsa05162:Measles	4.89228E−06	0.00022502
	KEGG_PATHWAY	hsa05164:Influenza A	2.9062E−06	0.000267335
	KEGG_PATHWAY	hsa05168:Herpes simplex infection	4.20496E−05	0.001288717
	GOTERM_MF_DIRECT	GO:0003725~double-stranded RNA binding	6.2164E−05	0.009466294
	GOTERM_BP_DIRECT	GO:0060333~interferon-gamma-mediated signaling pathway	0.000216281	0.024286767

Salmon	GOTERM_BP_DIRECT	GO:0030041~actin filament polymerization	0.000889415	0.183621158
	GOTERM_BP_DIRECT	GO:0007596~blood coagulation	0.001317229	0.139518478
	KEGG_PATHWAY	hsa04611:Platelet activation	0.003518509	0.179124611

### Biological Pathways in the PV-Associated Module “Salmon”

**Figure 4:**
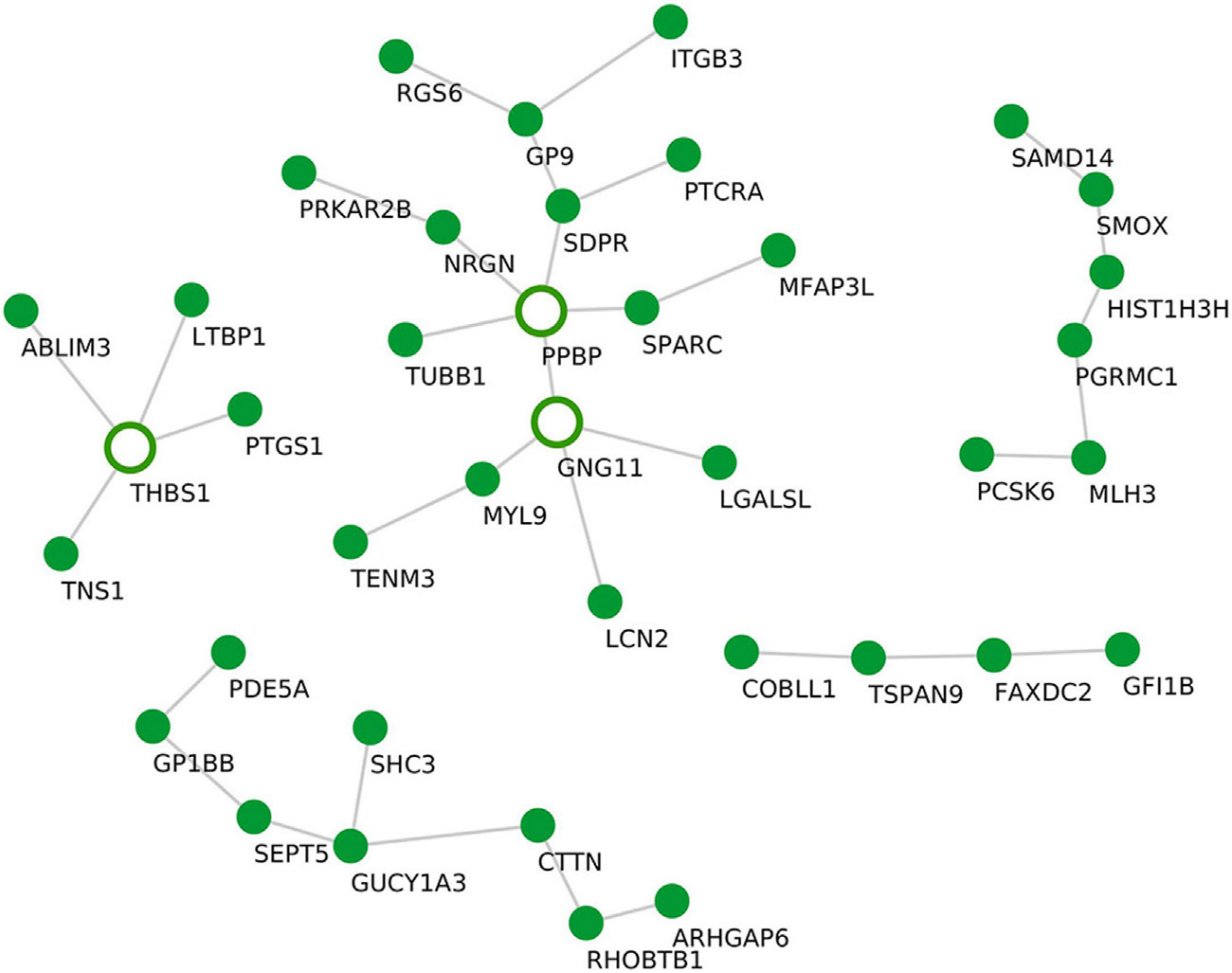
Gene–gene interaction network for the “salmon” module. *De novo* network generated by C3NET algorithm for the “salmon” module. The figure shows statistically significant (α < 0.05) edges predicted by the algorithm. Fully colored nodes represent the “salmon” module-associated genes. Empty nodes represent the regulatory genes (degree ≥ 4).

Although the sample size for PV samples was small (*n* = 4), we identified a distinct module that, compared with controls, was significantly upregulated in PV, namely the “salmon” module (*P* = 0.034). The “salmon” module comprises 39 genes (Table 1) and was enriched in the following biological processes: “blood coagulation” (P.adj = 1.4E−1) and the KEGG pathway “platelet activation” (P.adj = 1.8E−1). Using statistical module eigengenes, we identified platelet glycoprotein IX (GP9) as a hub gene of this module. Additionally, using the “c3net” algorithm, we identified pro-platelet basic protein (PPBP), G protein subunit gamma 11 (GNG11), and thrombospondin 1 (THBS1) genes as key regulators of the “salmon” module (degree ≥ 4) (Figure 4). In addition, while using the INMEX server we identified protein kinase cAMP-dependent type-II regulatory subunit beta (PRKAR2B), Src homology 2 domain-containing-transforming protein 3 (SHC3), tensin 1 (TNS1), PPBP, and GNG11 as regulatory genes (Figure S3 in Supplementary Material). Interestingly, both PPBP and GNG11 genes coincided with the list of the aforementioned C3NET-derived key regulatory genes.

### Cross-Linking SLE and Pemphigus GWA Studies with Clusters of Co-Expressed Genes in the “Magenta” and the “Salmon” Modules

**Figure 5:**
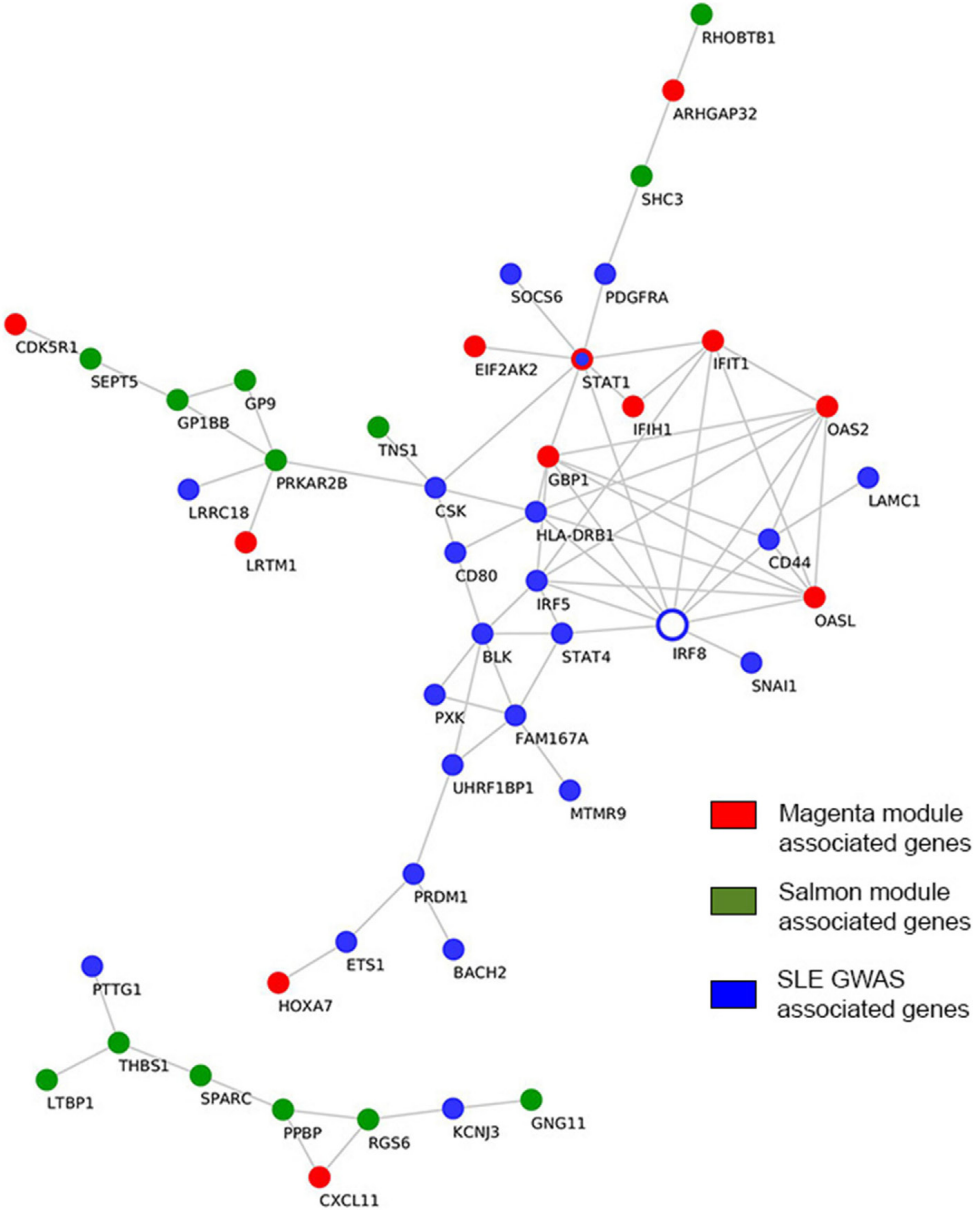
Interactions among genome-wide-associated genes and module-derived genes. Direct curated gene–gene interactions between modular genes and genes identified from SLE GWAS. Hub genes are represented by empty blue nodes. Common genes between SLE GWAS and the “magenta” module are denoted in blue nodes with red contour. SLE, systemic lupus erythematosus; GWAS, genome-wide association study.

While multiple GWA studies had been undertaken in a continuous effort to identify SLE susceptibility genes, only one GWA study was previously conducted in pemphigus, namely in PV (33, 34). In contrast to GWA studies that normally investigate the causal genes for a disease phenotype, gene expression profiles indicate the downstream effector phase. In the present work, we investigated direct interactions between previously reported susceptibility genes in SLE and pemphigus GWA studies and genes comprising the “magenta” and “salmon” modules, which were identified herein. We found the SLE-susceptible gene interferon regulatory factor 8 (IRF8) to have the largest number of direct interactions with “magenta” module-associated genes (Figure 5). The IRF8 gene interacted with genes encoding for interferon-induced protein with tetratricopeptide repeats 1 (IFIT1), interferon-induced guanylate-binding protein 1 (GBP1), 2’-5’-oligoadenylate synthetase 2 (OAS2), 2’-5’-oligoadenylate synthetase-like (OASL), and signal transducer and activator of transcription 1 (STAT1). Both IRF5 and STAT1 SLE GWAS genes directly interacted with IRF8 and with the other 4 “magenta” module-associated genes such as interferon induced with helicase C domain 1 (IFIH1), IFIT1, GBP1, OASL, OAS2, and EIF2AK2 (Figure 5). Polymorphism in the gene ST18 has been previously found in a PV GWA study. However, we could not identify direct interactions between ST18 and genes associated with the “salmon” module. To further establish a putative association of ST18 to other genes in the “salmon” module, we performed the transcriptional factor binding sites enrichment analysis (39 “salmon” genes and the ST18 gene). We observed that 34 out of the 40 analyzed genes are regulated by the nuclear hormone peroxisome proliferator activated receptor γ (PPAR-γ; P. adj = 8.3E−3) and 25 out of 40 genes are regulated by growth factor independent 1 transcriptional repressor (GFI1; P.adj = 8.3E−3).

## Discussion

The pathogenesis of most autoimmune disorders is still largely unknown. Environmental triggers in genetically susceptible individuals, as well as molecular mimicry mechanisms, may only partially account for this phenomenon (35). The co-occurrence of autoimmune diseases has been previously documented and aided in our understanding of autoimmunity (36).

Pemphigus and SLE are well-characterized autoimmune diseases that were previously reported to coexist in the same patient (37). Even though each of these two autoimmune diseases affects distinct organs and systems, the comorbidity of both diseases suggests an existence of fundamental common pathophysiological mechanisms. As we were interested in systems level similarity between the diseases rather than characterizing individual gene signatures, we used WGCNA to study pemphigus and SLE. Using this analytical approach, we identify modules across microarray datasets obtained from CD4^+^ T cells in pemphigus and SLE patients. In this study, we further demonstrate that gene expression data processed by two different batch correction algorithms remains biased and can lead to false positive estimations. Therefore, to standardize and remove batch effects from both datasets, we used “normexp,” “cyclicloess,” and “combat” algorithms. Using this strategy, we could compensate for the potential bias introduced by obtaining two distinct microarray datasets (Figure 1).

Our network analysis revealed two co-expression modules (denoted as “magenta” and “salmon”) that were significantly associated with PF and SLE, or PV only, respectively (Figure 2). Identification of the “magenta” module suggests common underlying mechanisms for pemphigus and SLE and identifies key regulatory genes for both diseases in CD4^+^ T cells. In terms of functional relevance, based on DAVID and KEGG ontology analyses, the “magenta” module is enriched in genes corresponding to type-I interferon (IFN) signaling and viral infection including herpes simplex, measles, and influenza viruses. Although type-I interferons were initially described and termed for their ability to “interfere” with viral replication, their role as immune modulators of both innate and adaptive immunity is now widely established (38). Moreover, a role for viruses in an induction of autoimmune diseases through several potential mechanisms, such as epitope spreading, molecular mimicry, cryptic antigens, and bystander activation, was also previously reported (39). The role of viral infection in the etiopathogenesis of SLE, the so-called “viral hypothesis,” has been extensively studied (40–42). SLE patients may present severe systemic viral infections primarily associated with Epstein-Bar virus (EBV), cytomegalovirus, and herpes simplex virus (HSV). With respect to pemphigus, in 1974, Krain et al. first reported the association between HSV and PV (43); meanwhile, several additional case reports were published examining this association (44–46). A more recent study by Kurata and colleagues demonstrated high levels of HSV DNA in the saliva of PV patients at the earliest stage of the disease without a history of herpetic infection, thus suggesting the presence of cases of pemphigus induced by herpesviruses (47).

In our work, on the basis of statistical module membership and its eigengene value, we identified RSAD2 gene as the hub gene of the “magenta” module. Notably, by examining the expression levels of RSAD2 gene in our datasets we could demonstrate its significant upregulation in PF (*P* = 0.005) and SLE (*P* = 0.007) in comparison to healthy controls (Figure S4A in Supplementary Material). To confirm, the expression of the RSAD2 gene is encoding for interferon-inducible viperin protein, which inhibits viral replication and facilitates T-cell receptor-mediated GATA3 activation, and optimal Th2 cytokine production through modulation of NFKB1 and JUNB activities. As a result, viperin-deficient mice show impaired Th2 cell development (48). Interestingly, transcripts for RSAD2 were found to be upregulated in SLE CD3^+^ CD4^+^ cells, as well as SLE CD19^+^ B cells, and SLE CD33^+^ myeloid cells in comparison to similar cellular subsets isolated from healthy controls (49). Although it has been previously demonstrated that Th2 cells exert broad activity in blister formation in pemphigus, the association of RSAD2 with pemphigus is unknown. To examine the relevance of Th2 response in pemphigus and SLE, a set of 44 genes associated with Th2 differentiation were downloaded from the PathCards Pathway Unification Database from the Weizmann Institute of Science, and examined for their fold change expression in our disease datasets (PV, PF, and SLE) in comparison to healthy controls (Figure S4B in Supplementary Material). Our findings confirm that the fold change expression of Th2-associated genes was positively correlated between SLE and PF (*P* = 0.01, ρ = 0.36) and between SLE vs. PV (*P* = 1.087E−05 ρ = 0.62), suggesting that the Th2 response is skewed in a similar pattern between SLE and pemphigus. While investigating subnetworks within the “magenta” module (using the “c3net” algorithm), we identified OAS1, MX1, IFIT3, and SPATS2L genes as master regulators (Figure 3). Additional regulatory genes such as JUP, BCL2, ISG15, STAT1, SKP2, and EIF2AK2 were identified using known gene–gene interactions database (INMEX) (Figure S2 in Supplementary Material). Transcripts of 7 out of the 11 identified genes (i.e., RSAD2, OAS1, MX1, IFIT3, ISG15, STAT1, and EIF2AK2) were previously shown to be upregulated in SLE CD3^+^ CD4^+^ cells (49). Consistent with a previous study that examined possible related signaling pathways shared in the pathogenesis of several systemic autoimmune diseases (SAID) such as dermatomyositis, polymyositis, rheumatoid arthritis, and SLE, a subset of five viral-related differentially expressed genes (i.e., RSAD2, IFIT3, ISG15, STAT1, and EIF2AK) was detected in peripheral blood of SAID probands and their unaffected twins (50). Additionally, other genes that were identified in our study, including BCL2, OAS1, MX1, and SKP2 have been previously associated with various autoimmune diseases (51–54). Therefore, our findings further suggest that these common IFN signature genes are shared across multiple autoimmune diseases including pemphigus and SLE.

Here, we identified a PV-specific associated module. The “salmon” module consisted of 39 genes and was enriched in genes involved in blood coagulation and platelet activation. Based on the eigenegene value, the gene GP9 was identified as the hub gene of the “salmon” module. GP9 encodes a small-membrane glycoprotein that is part of the GPIb-V-IX complex that mediates platelet adhesion to blood vessels and promotes hemostasis. Thus, mutations in the GP9 protein lead to a coagulation disorder, also known as the Bernard–Soulier syndrome, characterized by thrombocytopenia. Of note, although this is a first report suggesting a role for GP9 in PV, a previous study by Hunziker et al. identified platelet-derived factors to enhance pemphigus acantholysis in skin organ cultures (55). Moreover, another study by Mizutani et al. found increased expression of the coagulation factor on keratinocytes, which shield blisters in PV (56). In line with this observation, using the “c3net” algorithm, we identified an additional list of platelet-associated genes i.e., PPBP, GNG11, and THSB1, as key regulators of the “salmon” module (Figure 4). Furthermore, by examining known gene–gene interactions, we could identify PPBP, GNG11, as well as another group of platelet-function-associated genes such as PRKAR2B, SHC3, and TNS1 (Figure S3 in Supplementary Material) as additional regulators of this module.

Further in our analysis, we associated the genes found in the “magenta” and “salmon” modules with known susceptibility markers of PV and SLE, which had been formerly identified by GWASs. GWASs are applied to identify genetic variants that are associated with a disease trait. However, the identification of loci harboring the susceptible genes does not fully reveal the molecular mechanisms that are at play to yield the observed phenotype. Therefore, linking these susceptibility genes with the module-associated genes may identify pathways that control the disease phenotype and provide potential therapeutic targets for intervention. By cross-linking susceptibility genes derived from SLE GWAS with clusters of co-expressed genes in “magenta” module, we found IRF8 to directly interact with the largest number of interferon-induced genes present in the “magenta” module including IFIT1, GBP1, OAS2, OASL, and STAT1 (Figure 5). Interestingly, STAT1 was identified both as an SLE susceptibility gene and as a key regulator gene of the “magenta” module. Therefore, based on our analysis, we predict IRF8 to have pharmacological relevance, as previously described (57). With regard to PV, we did not identify direct interactions between the known GWAS gene, ST18, and the 39 “salmon” module-associated genes. To circumvent this finding, we additionally performed a transcriptional factor binding sites enrichment analysis for the 40 genes. We found that the majority of the genes are regulated by the transcription factors PPAR-γ and GFI1 that have been previously described for their role in Th2 cell development (58, 59). Moreover, PPAR-γ has been suggested as a pharmacological target for PV (60).

Altogether, our work reveals conserved molecular mechanisms and pathways between pemphigus and SLE and identifies novel gene candidates that could be used as biomarkers or as potential targets for therapeutic intervention.

## Author Contributions

TS, AV, YG, and RL designed the study, interpreted the data, and wrote the manuscript. All authors contributed equally to this work. YG downloaded and analyzed the data. CS and DZ discussed the results and contributed to the writing of the manuscript.

## Conflict of Interest Statement

The authors declare that the research was conducted in the absence of any commercial or financial relationships that could be construed as a potential conflict of interest.
