# Supplementary material for: Gene Expression Analysis Reveals Novel Shared Gene Signatures and Candidate Molecular Mechanisms between Pemphigus and Systemic Lupus Erythematosus in CD4+ T Cells
Source: Front Immunol. 2018 Jan 17;8:1992. doi: 10.3389/fimmu.2017.01992 (PMC5776326; doi:10.3389/fimmu.2017.01992)
Supplement: Supplementary file 1 [file Image_1.PDF]

Supplementary data to:
Gene Expression Analysis Reveals Novel Shared Gene Signatures and Candidate Molecular Mechanisms Between Pemphigus and Systemic Lupus Erythematosus in CD4^{+} T cells
by Tanya Sezin, Artem Vorobyev, Christian D. Sadik, Detlef Zillikens, Yask Gupta and Ralf J. Ludwig

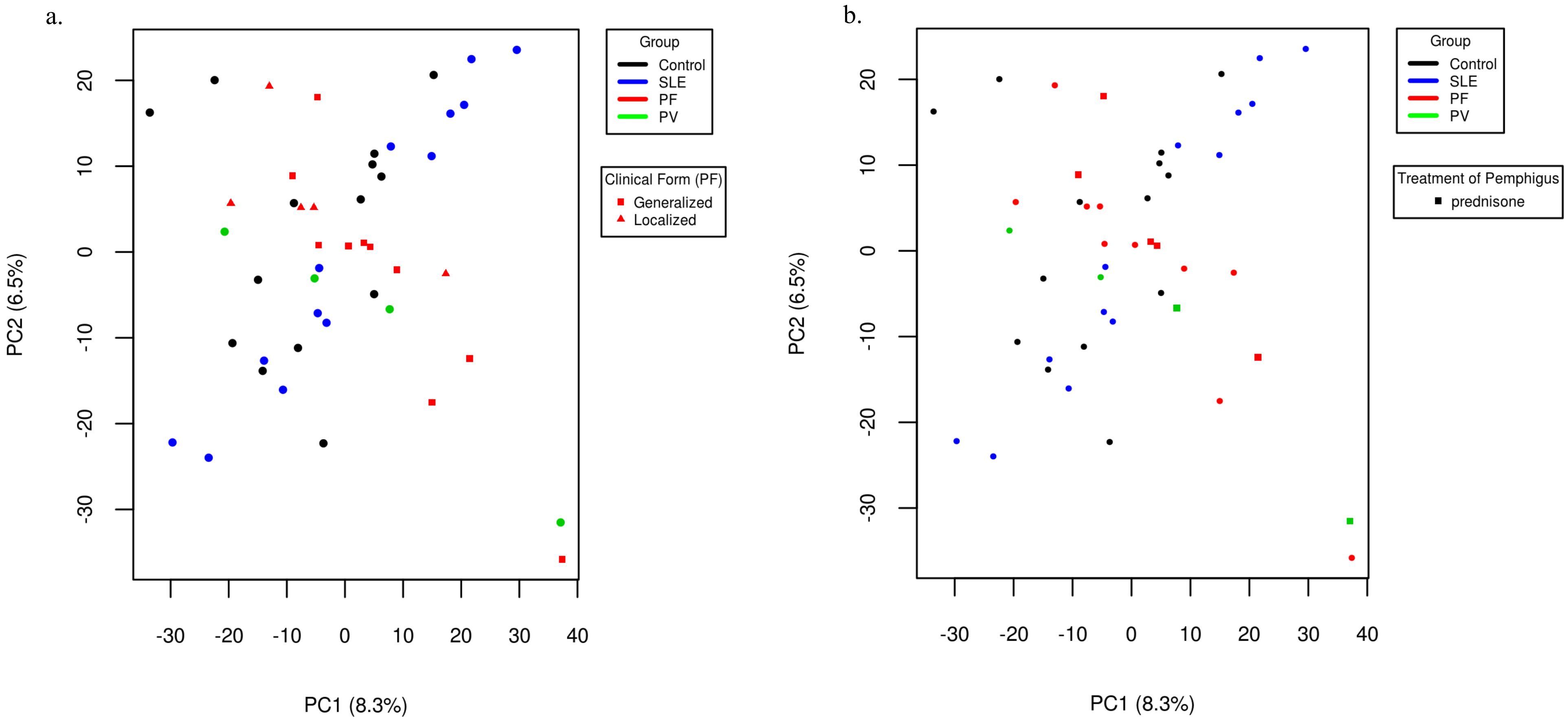

Supplementary Figure 1. PCA plots illustrating dataset stratification based on clinical form (a) and treatment (b). The X- and Y-axes represent the 1st and the 2nd principal components and the associated percentage of variation.
