# Supplementary material for: Gene Expression Analysis Reveals Novel Shared Gene Signatures and Candidate Molecular Mechanisms between Pemphigus and Systemic Lupus Erythematosus in CD4+ T Cells
Source: Front Immunol. 2018 Jan 17;8:1992. doi: 10.3389/fimmu.2017.01992 (PMC5776326; doi:10.3389/fimmu.2017.01992)
Supplement: Supplementary file 3 [file Image_3.PDF]

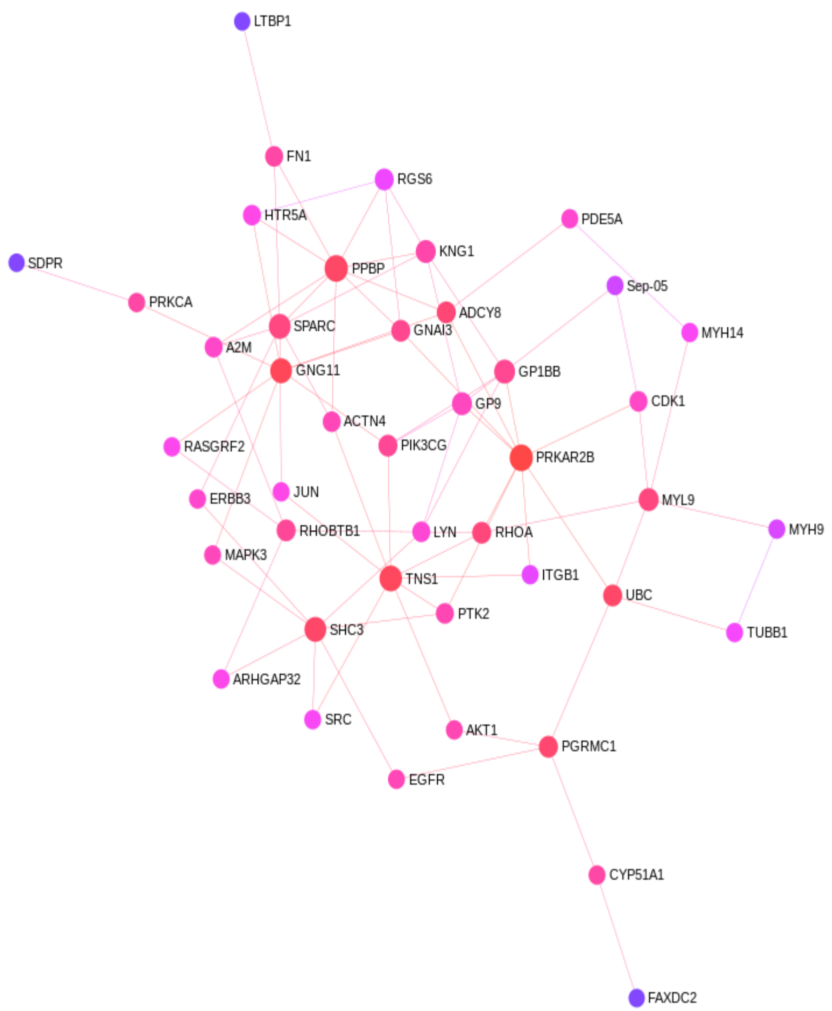


**Supplementary Figure 3. Known gene-gene interactions predicted by the INMEX web server for the PV-associated "salmon" module.** The key genes are shown in red
