# Supplementary material for: Gene Expression Analysis Reveals Novel Shared Gene Signatures and Candidate Molecular Mechanisms between Pemphigus and Systemic Lupus Erythematosus in CD4+ T Cells
Source: Front Immunol. 2018 Jan 17;8:1992. doi: 10.3389/fimmu.2017.01992 (PMC5776326; doi:10.3389/fimmu.2017.01992)
Supplement: Supplementary file 4 [file Image_4.PDF]

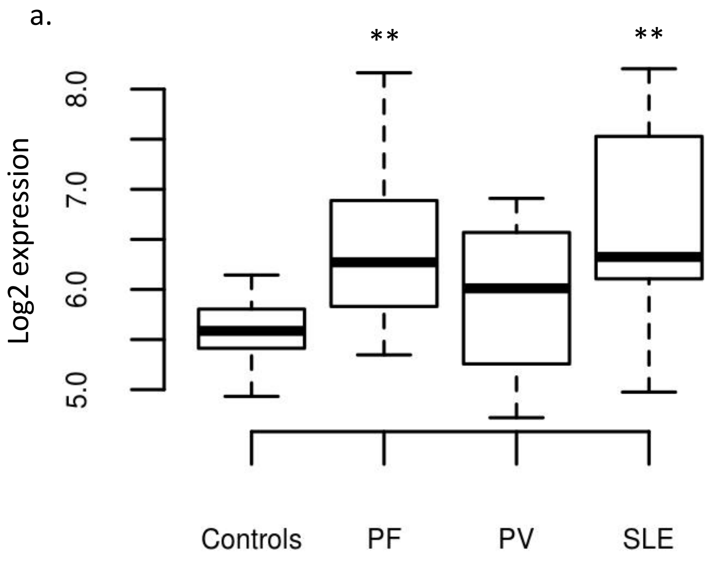

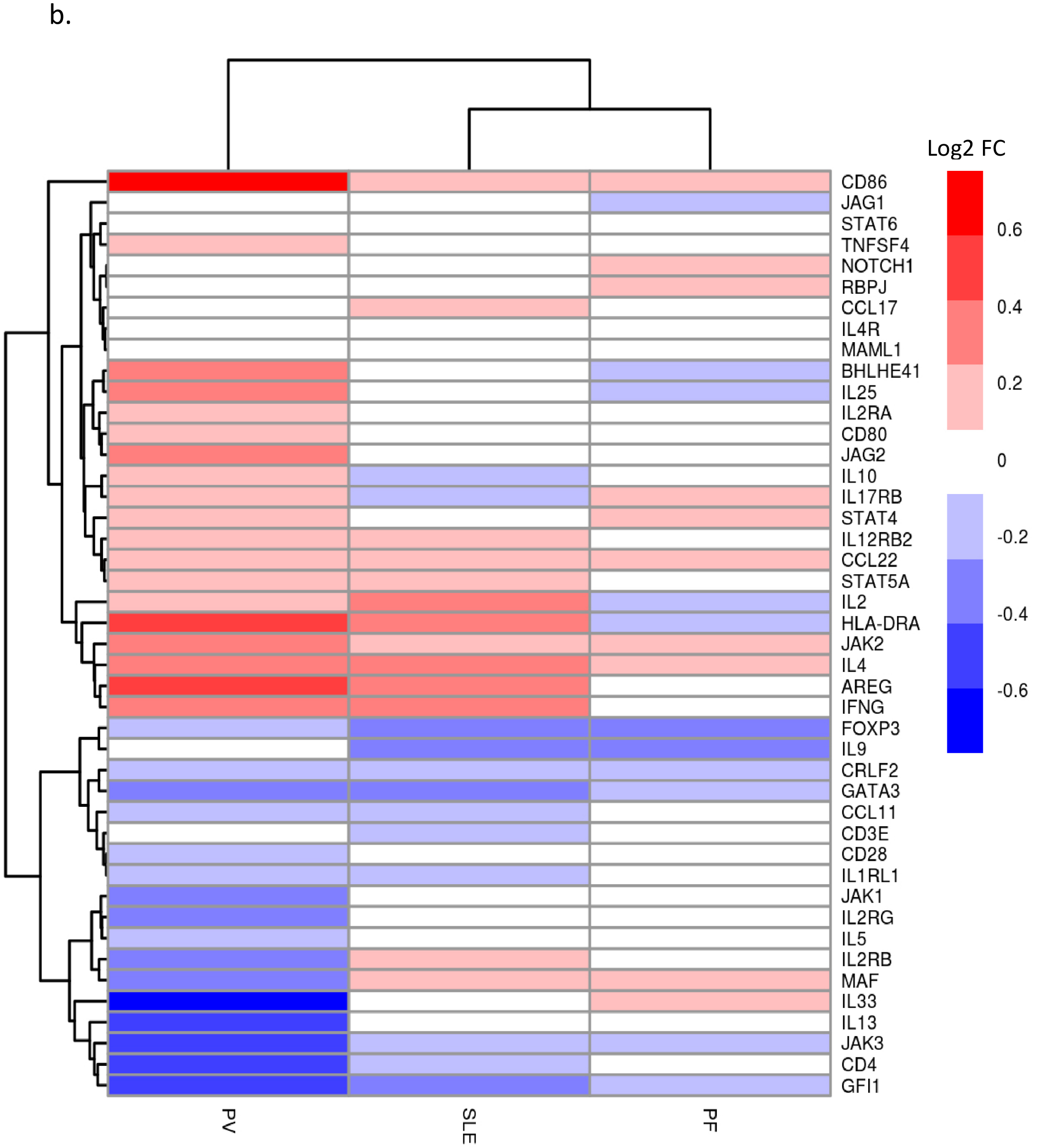

Supplementary Figure 4. (a) Boxplot illustrating expression levels of RSAD2 in pemphigus (GSE53873) and SLE (GDS4185) vs controls. The significance among the groups was calculated using Kruskal-Wallis test followed by a Mann-Whitney U post hoc test. The values were adjusted for multiple comparisons using FDR correction. $^{**}P < 0.01$;
(b) Heatmap depicting modulation of Th2-associated genes in pemphigus (GSE53873) and SLE (GDS4185). The heatmap shows fold change of Th2-associated genes (subset mentioned in PathCards Pathway Unification Database, Weizmann Institute of Science) expression levels in disease vs controls. Upregulated genes are indicated by the red boxes; downregulated genes by blue boxes, and non-differentially expressed genes by white boxes. PF: pemphigus foliaceus; PV: pemphigus vulgaris; SLE: systemic lupus erythematosus; FC: fold change.
