# Supplementary material for: Gene Expression Analysis Reveals Novel Shared Gene Signatures and Candidate Molecular Mechanisms between Pemphigus and Systemic Lupus Erythematosus in CD4+ T Cells
Source: Front Immunol. 2018 Jan 17;8:1992. doi: 10.3389/fimmu.2017.01992 (PMC5776326; doi:10.3389/fimmu.2017.01992)
Supplement: Supplementary file 5 [file Table_1.DOCX]

| **Accession No.** | **Sample ID** | **Disease** | **Clinical form** | **Age** | **Sex** | **Treatment** |
| --- | --- | --- | --- | --- | --- | --- |
| GSE53873 | GSM1302416 | PF | Localized | 21 | Female | none |
| GSE53873 | GSM1302417 | PF | Generalized | 29 | Female | prednisone |
| GSE53873 | GSM1302418 | PF | Localized | 37 | Female | none |
| GSE53873 | GSM1302419 | PF | Generalized | 27 | Female | none |
| GSE53873 | GSM1302420 | PF | Generalized | 15 | Male | none |
| GSE53873 | GSM1302421 | PF | Localized | 51 | Female | none |
| GSE53873 | GSM1302422 | PF | Localized | 26 | Female | none |
| GSE53873 | GSM1302423 | PF | Generalized | 39 | Female | prednisone |
| GSE53873 | GSM1302424 | PF | Generalized | 33 | Male | none |
| GSE53873 | GSM1302425 | PF | Generalized | 12 | Male | prednisone |
| GSE53873 | GSM1302426 | PF | Generalized | 41 | Female | none |
| GSE53873 | GSM1302427 | PF | Localized | 32 | Female | none |
| GSE53873 | GSM1302428 | PF | Generalized | 45 | Male | prednisone |
| GSE53873 | GSM1302429 | PF | Generalized | 29 | Female | prednisone |
| GSE53873 | GSM1302430 | PF | Generalized | 33 | Female | none |
| GSE53873 | GSM1302431 | PV | Mucocutaneous | 52 | Female | none |
| GSE53873 | GSM1302432 | PV | Mucocutaneous | 48 | Female | prednisone |
| GSE53873 | GSM1302433 | PV | Mucocutaneous | 42 | Female | none |
| GSE53873 | GSM1302434 | PV | Mucosal | 24 | Female | prednisone |
| GSE53873 | GSM1302435 | Control | Control | 30 | Male | none |
| GSE53873 | GSM1302436 | Control | Control | 52 | Male | none |
| GSE53873 | GSM1302437 | Control | Control | 44 | Male | none |
| GSE53873 | GSM1302438 | Control | Control | 33 | Male | none |
| GSE53873 | GSM1302439 | Control | Control | 36 | Female | none |
| GDS4185 | GSM260886 | Control | Control | NA | NA | NA |
| GDS4185 | GSM260889 | Control | Control | NA | NA | NA |
| GDS4185 | GSM260891 | Control | Control | NA | NA | NA |
| GDS4185 | GSM260894 | Control | Control | NA | NA | NA |
| GDS4185 | GSM260897 | Control | Control | NA | NA | NA |
| GDS4185 | GSM260900 | Control | Control | NA | NA | NA |
| GDS4185 | GSM260903 | Control | Control | NA | NA | NA |
| GDS4185 | GSM260906 | Control | Control | NA | NA | NA |
| GDS4185 | GSM260909 | Control | Control | NA | NA | NA |
| GDS4185 | GSM260914 | SLE | NA | NA | NA | NA |
| GDS4185 | GSM260916 | SLE | NA | NA | NA | NA |
| GDS4185 | GSM260919 | SLE | NA | NA | NA | NA |
| GDS4185 | GSM260922 | SLE | NA | NA | NA | NA |
| GDS4185 | GSM260925 | SLE | NA | NA | NA | NA |
| GDS4185 | GSM260927 | SLE | NA | NA | NA | NA |
| GDS4185 | GSM260930 | SLE | NA | NA | NA | NA |
| GDS4185 | GSM260933 | SLE | NA | NA | NA | NA |
| GDS4185 | GSM260936 | SLE | NA | NA | NA | NA |
| GDS4185 | GSM260939 | SLE | NA | NA | NA | NA |
| GDS4185 | GSM260942 | SLE | NA | NA | NA | NA |
| GDS4185 | GSM260945 | SLE | NA | NA | NA | NA |
| GDS4185 | GSM260950 | SLE | NA | NA | NA | NA |
